# FANCC deficiency mediates microglial pyroptosis and secondary neuronal apoptosis in spinal cord contusion

**DOI:** 10.1186/s13578-022-00816-4

**Published:** 2022-06-03

**Authors:** Mingjie Xia, Xinyu Li, Suhui Ye, Qinyang Zhang, Tianyu Zhao, Rulin Li, Yanan Zhang, Minghan Xian, Tianqi Li, Haijun Li, Xin Hong, Shengnai Zheng, Zhanyang Qian, Lei Yang

**Affiliations:** 1 Department of Orthopedics, Nanjing First Hospital, Nanjing Medical University, 68 Changle Road, Nanjing, 210029 Jiangsu China; 2 School of Health Economics Management, Nanjing University of Chinese Medicine, Nanjing, China; 3 Spine Center, Zhongda Hospital of Southeast University, Nanjing, China; 4 School of Medicine, Southeast University, Nanjing, China; 5 Postgraduate School, Dalian Medical University, Dalian, China; 6 Department of Orthopedics, Taizhou People’s Hospital, Nanjing Medical University, No. 366 Taihu Road, Taizhou, 225300 Jiangsu China; 7 School of Biomedical Engineering and Informatics, Nanjing Medical University, Nanjing, China

**Keywords:** Spinal cord injury, FANCC, NLRP3, Microglia, Pyroptosis, Neuronal apoptosis

## Abstract

**Background:**

Traumatic spinal cord injury (SCI)-induced neuroinflammation results in secondary neurological destruction and functional disorder. Previous findings showed that microglial pyroptosis plays a crucial role in neuroinflammation. Thus, it is necessary to conduct a comprehensive investigation of the mechanisms associated with post-SCI microglial pyroptosis. The Fanconi Anemia Group C complementation group gene (FANCC) has been previously reported to have an anti-inflammation effect; however, whether it can regulate microglial pyroptosis remains unknown. Therefore, we probed the mechanism associated with FANCC-mediated microglial pyroptosis and neuroinflammation in vitro and in vivo in SCI mice.

**Methods:**

Microglial pyroptosis was assessed by western blotting (WB) and immunofluorescence (IF), whereas microglial-induced neuroinflammation was evaluated by WB, Enzyme-linked immunosorbent assays and IF. Besides, flow cytometry, TdT-mediated dUTP Nick-End Labeling staining and WB were employed to examine the level of neuronal apoptosis. Morphological changes in neurons were assessed by hematoxylin–eosin and Luxol Fast Blue staining. Finally, locomotor function rehabilitation was analyzed using the Basso Mouse Scale and Louisville Swim Scale.

**Results:**

Overexpression of FANCC suppressed microglial pyroptosis via inhibiting p38/NLRP3 expression, which in turn reduced neuronal apoptosis. By contrast, knockdown of FANCC increased the degree of neuronal apoptosis by aggravating microglial pyroptosis. Besides, increased glial scar formation, severe myelin sheath destruction and poor axon outgrowth were observed in the mice transfected with short hairpin RNA of FANCC post SCI, which caused reduced locomotor function recovery.

**Conclusions:**

Taken together, a previously unknown role of FANCC was identified in SCI, where its deficiency led to microglia pyroptosis, neuronal apoptosis and neurological damage. Mechanistically, FANCC mediated microglia pyroptosis and the inflammatory response via regulating the p38/NLRP3 pathway.

**Supplementary Information:**

The online version contains supplementary material available at 10.1186/s13578-022-00816-4.

## Introduction

Spinal cord injury (SCI) is a devastating disease of the central nervous system (CNS) leading to paralysis and even death, which affects patients’ quality of life and results in a heavy financial burden on their families [1–3]. The pathogenesis of SCI is complicated, in that the initial trauma (primary SCI) causes primary neural injuries that then result in a stream of neurological sequelae (secondary SCI), which severely affect neurologic functional recovery over an extended period of time [4, 5]. As the resident macrophages of the CNS, resting microglia rapidly activate in response to primary SCI and release a variety of proinflammatory cytokines and mediators that evoke neuroinflammation in the neural lesion and contribute to the biological cascades during secondary SCI [6, 7]. Pyroptosis, also known as inflammatory necrosis, is a modality of programmed cell death mediated via multifarious inflammation-related signaling pathways [8–10]. Specifically, after cellular inflammation has been initiated, the activation of inflammasomes containing apoptosis-associated speck-like protein containing CARD (ASC), caspase proteases and Nod-like receptor (NLR) family proteins, cleave the precursor of caspase-1, which further promotes the formation of active N-terminus pore-forming protein gasdermin D (GSDMD-N), interleukin-1β (IL-1β) and IL-18, leading to pyroptosis that amplifies inflammation [11–13]. Previous studies confirmed that microglial pyroptosis is involved in the inflammatory process and that blocking it attenuates neuroinflammation levels and neuronal apoptosis post SCI [14, 15]. However, the underlying molecular mechanism of post-SCI microglial pyroptosis still remains unclear.

The Fanconi Anemia Group C complementation group gene (FANCC) encodes a protein, FANCC, which plays an indispensable role in the pathogenesis of Fanconi Anemia [16–18]. Besides, FANCC was reported to regulate bone marrow failure pathogenesis in human inherited bone marrow failure syndromes [19]. Although the mechanism of FANCC was elaborated in hematological diseases, there has been little research concerning FANCC in inflammatory diseases since Hadjur et al. first found that FANCC-deficient mice were more sensitive to proinflammatory factors like IL-1β and tumor necrosis factor-α [20]. Given the results of the previous study, we speculated that FANCC may participate in microglia-induced neuroinflammation and regulate IL-1β-related pyroptosis signaling post SCI.

Here, we identified a previously unidentified role of FANCC in microglial pyroptosis and the subsequent neuroinflammatory response post SCI. We utilized lipopolysaccharide (LPS) to mimic SCI-induced microglial inflammation to trace the specific molecular mechanism associated with FANCC-mediated regulation of microglial pyroptosis, established microglia-neuron co-cultures in vitro to clarify the effect of FANCC on inflammatory microglia-induced neuronal apoptosis and employed silencing of FANCC in SCI mice via short hairpin (sh) RNA to demonstrate the neuroprotective potential of FANCC in SCI mice.

## Methods and materials

### Isolation and culture of primary cells

One-day-old mice were sacrificed in 75% ethanol, the brains removed to precooled Dulbecco’s modified Eagle medium (DMEM; KeyGEN, Nanjing, China) and the meninges and blood vessels removed. The cerebral cortex was then isolated, placed in new DMEM, cut into pieces and 2 mg/mL papainase (Sigma-Aldrich, St. Louis, MO, USA) added. The cortical tissue was then shaken on a shaker for 10 min at a constant temperature of 37 °C. After centrifugation at 1200 g for 5 min, the supernatant was removed and cells were resuspended and incubated in DMEM containing 10% fetal bovine serum (FBS; Gibco, Grand Island, NY, USA) at 37 °C. For neuronal isolation, the cells were resuspended in neurobasal medium (Gibco) after 4 h cultured, then the medium was changed every 2 days. For microglia isolation, the medium was changed every 3 days. After 14 days, microglial cultures were shaken on a shaker for 4 h at a constant temperature of 37 °C and the cells collected in 6-well plates (Corning, NY, USA) containing DMEM containing 10% FBS. Microglia were pretreated with the p38 MAPK inhibitor BIRB 796 (500 nM, Axon Medchem, Groningen, The Netherlands) for 6 h or the NLRP3 inhibitor glyburide (50 mM, Sigma-Aldrich) for 2 h, after which LPS (1 μg/mL, Sigma-Aldrich) was administered to activate microglial inflammation for 24 h.

### Co-culture of microglia and neurons

To mimic the effect of microglial inflammation on neuronal apoptosis in vitro, a co-culture model was established as shown in Fig. 3A. Neurons (in 500 μL of neurobasal medium) were incubated on the bottom of the 24-well plates (Corning) for 24 h, after which cell inserts (Corning) were placed in each well and the treated microglia, suspended in 200 μL medium, were seeded on the top of the inserts and cultured for 72 h.

### SCI model

C57BL/6 J adult mice, (males, average weight of 20 g, 8 weeks of age) purchased from Charles River (Beijing, China), were anesthetized with ketamine (80 mg/kg). The SCI protocol used here was the same as that in our previous study [21]. Briefly, the skin was cut and the fascia muscle separated on the back, after which the lamina at T10 segment was resected. A moderate contusion (5 g × 5 cm) was created on the spinal cord of the mouse using a spinal cord impactor (RWD, Shenzhen, China). The SCI mice were treated with artificial micturition twice per day until their cystospasm was eliminated. The sham mice received a T10 laminectomy only.

### Regulation of FANCC expression

The FANCC overexpression (OE-FANCC) and shRNA targeting FANCC for knockdown (KD-FANCC) were loaded in plasmids, which were constructed from BIOG CO., LTD (Changzhou, China), and respectively transfected into primary microglial cultures using RFect Plasmid Transfection Reagent (BIOG). The shRNA-FANCC adeno-associated virus (AAV) vector, which was injected into the tail veins of mice at 1 week before SCI modeling, was obtained from Genechem CO., LTD (Shanghai, China).

### Real-time quantitative reverse-transcription PCR (qRT-PCR)

Total RNA from microglia and spinal cords was extracted using TRIzol reagent (YiFeiXue Biotechnology, Nanjing, China) according to the manufacturer's instructions. Reverse transcription was performed using the Yfx 1st Strand cDNA Synthesis Kit (YiFeiXue Biotechnology). Quantitative analysis of RNA was accomplished using the Roche LightCycler 480 (Roche, Basel, Switzerland) with a qPCR Kit (YiFeiXue Biotechnology). The primer sequences were as follows: FANCC: forward: GAGACAGGACTTAACTCGTGGA; reverse: AGCCATCCGACTTTGAGTGC; GAPDH: forward: TGACCTCAACTACATGGTCTACA; reverse: CTTCCCATTCTCGGCCTTG.

### Western blotting (WB) assay

Total protein was extracted from cells and cord tissue using the Total Protein Extraction Kit (KeyGEN) according to the manufacturer’s instructions, after which the concentration was quantified using the Enhanced BCA Protein Assay Kit (Beyotime, Shanghai, China). Total of 60 μg proteins in each group were used for WB. Relevant primary and secondary antibodies used in WB analyses were as follows: anti-FANCC (1:1000, 22,857, Singalway Antibody, CollegePark, MD, USA), anti-p38 (1:1000, 14,451, Cell Signaling Technology, Boston, MA, USA), anti-phospho-p38 (1:1000, 4092, Cell Signaling Technology), anti-NLRP3 (1:1000, 29,125, Singalway Antibody), anti-ASC (1:1000, 40,618, Singalway Antibody), anti-GSDMD (1:1000, ab219800, Abcam, Cambridge, MA, USA), anti-GSDMD-N (1:1000, ab215203, Abcam), anti-Caspase-1 (1:1000, ab138483, Abcam), anti-cleaved-Caspase-1(1:1000, 89,332, Cell Signaling Technology), anti-IL-1β (1:1000, 12,703, Cell Signaling Technology), anti-cleaved-IL-1β (1:1000, 83,186, Cell Signaling Technology), anti-β-actin (1:10,000, HRP-60008, Proteintech, Rosemount, IL, USA), anti-β-tubulin (1:10,000, HRP-66240, Proteintech), HRP Goat-anti-Rabbit secondary antibody (1:10,000, YFSA02, YiFeiXue Biotechnology) and HRP Goat-anti-Mouse secondary antibody (1:10,000, YFSA01, YiFeiXue Biotechnology). Protein signals were captured with a Gel Document System (SYNGENE, Cambridge, UK), followed by quantitative analysis using ImageJ software (National Institutes of Health, Bethesda, MD, USA).

### Enzyme-linked immunosorbent assays (ELISAs)

Cord tissues (5 mm length) were minced into homogenate and centrifuged at 4 °C, after which the supernatants were stored at − 80 °C until further use. ELISAs were performed to test the levels of IL-1β and IL-18 in supernatants using respective ELISA Kits (YiFeiXue Biotechnology) according to the manufacturer's instructions. The absorbance was then determined at 450 nm using a microplate reader (BioTek, Vermont, USA).

### Flow cytometry assay (FCA)

The degree of neuronal apoptosis was detected using the Apoptosis Detection Kit (YiFeiXue Biotechnology) according to manufacturer’s instructions. Neurons were incubated with Annexin V-FITC reagent at room temperature for 10 min, followed by incubation with Propidium Iodide reagent for 5 min. Primary microglia were incubated with F4_80-PE (565,410, BD Biosciences, Franklin Lakes, NJ, USA) and iNOS-FITC (610,330, BD Biosciences) for 30 min at 4 °C, injected into a flow cytometer (FACSVerse 8, BD) and the obtained data were analyzed using FlowJo software (Version 7.6.1; FlowJo, Treestar, OR, USA).

### TUNEL staining

Neuronal and microglial death was measured using a TUNEL Staining Kit (Servicebio, Wuhan, China) according to the manufacturer’s instructions. Briefly, neurons and sections of spinal cords were incubated with antigen retrieval solution prior to permeabilization, treated with a mixture of terminal deoxynucleotidyl transferase enzyme, deoxyuridine triphosphates and buffer at ratio of 1:5:50, then were counterstained with diaminobenzidine (DAPI) to visualize the nuclei and observed under a fluorescence microscope (Leica, Oskar, Germany).

### Immunofluorescence (IF) staining

After blocking with Immunol Staining Blocking Buffer (Beyotime) for 1 h, sections and cells were incubated with primary antibodies overnight at 4 °C, followed by incubation with fluorescent secondary antibodies in the dark for 1 h. The antibodies used were as follows: anti-FANCC (1:100, 22,857, Singalway Antibody), anti-NeuN (1:100, MAB377; Millipore, Burlington, MA, USA), anti-GFAP (1:300, 3670; Cell Signaling Technology), anti-IBA-1 (1:500, ab178847; Abcam), anti-NLRP3 (1:100, 29,125, Singalway Antibody), anti-iNOS (1:100, ab15323, Abcam), anti-Caspase-1 (1:100, ab138483, Abcam) and anti-NF200 (1:200, ab82259, Abcam). After counterstaining with DAPI, the samples were visualized under a fluorescent microscope (Leica). The fluorescence intensity in the images was analyzed using ImageJ software (National Institutes of Health).

### Nissl, hematoxylin–eosin (HE) and Luxol Fast Blue (LFB) staining

To evaluate the number of neurons, morphology of the spinal cord tissue and integrity of the neuronal myelin sheath after SCI, we used Nissl Staining Reagent (Servicebio), HE Staining Reagent (Servicebio) and LFB Staining Reagent (Servicebio), respectively, following the manufacturer’s instructions. The sections were under an optical microscope (Leica).

### Behavioral assessment

Locomotor function of hindlimbs was evaluated using the Basso Mouse Scale (BMS) and Louisville Swim Scale (LSS) as previously described [22, 23]. Mice in each group were tested in an open field assessed by two blinded researchists at 1, 3, 7, 14, 21 and 28 days post injury (dpi).

### Statistical analysis

Data are shown as the mean ± standard deviation values and were analyzed using Prism software, version 8.3 (GraphPad, San Diego, CA, USA). Comparisons between two groups were analyzed using t-tests and among more than two groups using one-way or two-way ANOVAs followed by Tukey’s post hoc test. *P*-value < 0.05 were considered as significance.

## Results

### FANCC deficiency induces microglial pyroptosis by regulating p38-MAPK and inflammasome NLRP3

**Fig. 1:**
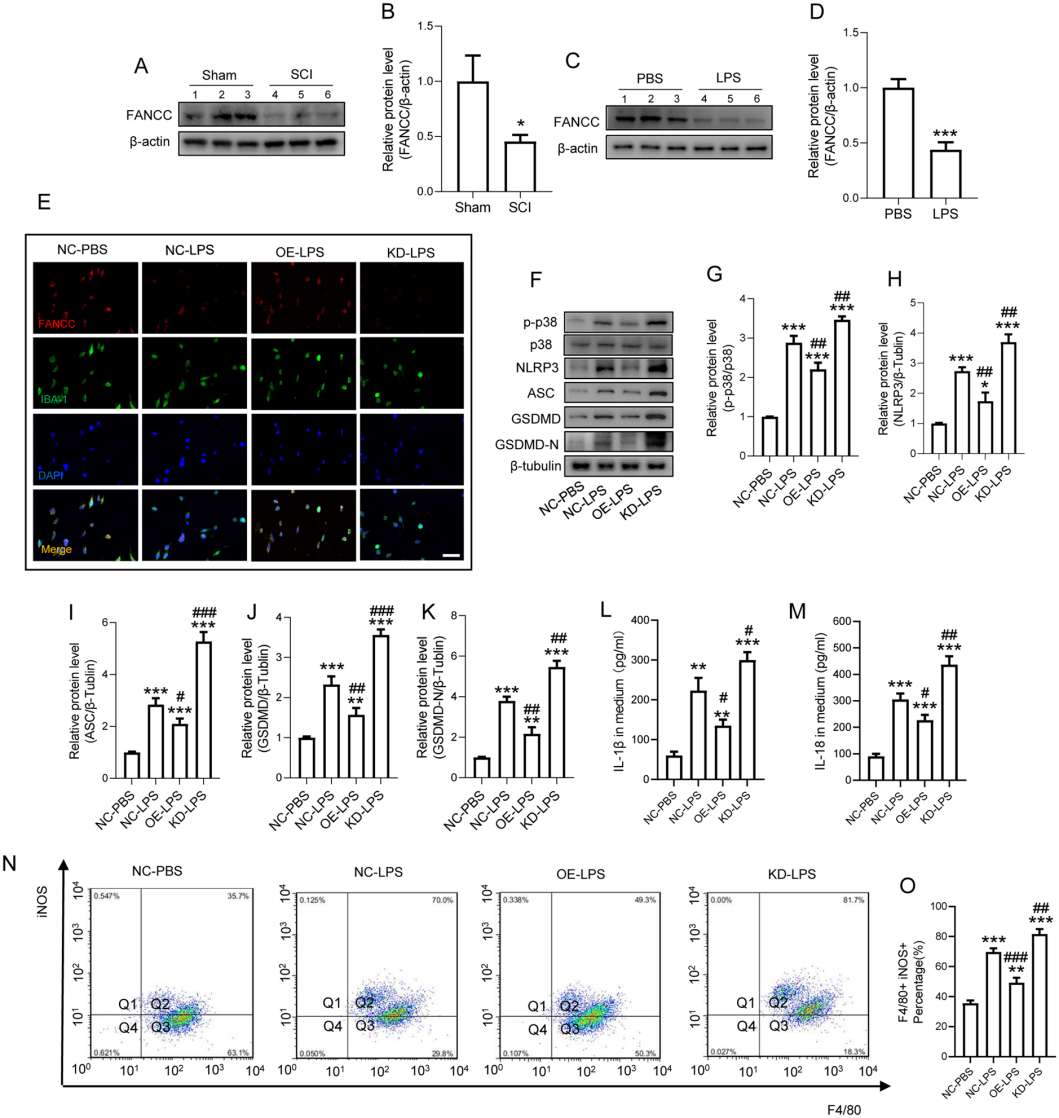
FANCC deficiency induces microglial pyroptosis by regulating p38-MAPK and inflammasome NLRP3. **A** Western blotting of FANCC protein levels in the spinal cord at 7 days post-injury; n = 3. **B** Bar graph showing a quantitative analysis of FANCC expression; n = 3. The error bars represent the SD. *p < 0.05 vs. Sham group by t-test (*p < 0.05, **p < 0.01, and ***p < 0.001). **C** Western blotting of FANCC in LPS-stimulated primary microglia for 24 h; n = 3. β-actin was used as the control. **D** Bar graph showing the densitometry analysis of FANCC expression. The error bars represent the SD. *p < 0.05 vs. PBS group by t-test (*p < 0.05, **p < 0.01, and ***p < 0.001). **E** Representative immunofluorescence labeling images for IBA-1 (green) and FANCC (red) in LPS-activated microglia after transfection with OE-FANCC and KD-FANCC; Scale bar = 50 μm. **F** Western blotting for p-p38, p38, NLRP3, ASC, GSDMD and GSDMD-N in LPS-activated microglia after transfection with OE-FANCC and KD-FANCC; n = 3. **G** Bar graph showing the ratio analysis of p-p38/p38. **H** Densitometric analysis of NLRP3 expression. **I** Densitometric analysis of ASC expression. **J** Densitometric analysis of GSDMD expression. **K** Densitometric analysis of GSDMD-N expression. **L**, **M** ELISAs performed for the IL-1β and IL-18 in culture medium obtained at 24 h in LPS-activated microglia after transfection with OE-FANCC and KD-FANCC (n = 5). **N** Representative flow cytometry performed for the distinction of iNOS^+^&F4/80^+^ microglia transfected OE-FANCC and KD-FANCC post 24 h LPS inducement. **O** Bar graph showing the quantitative analysis of the percent; n = 4. The error bars represent the SD. *p < 0.05 vs. NC-PBS group, #p < 0.05 vs. NC-LPS group by one-way ANOVA followed by Tukey’s post hoc analysis (*p < 0.05, **p < 0.01, and ***p < 0.001). *NC-PBS* microglia were treated with PBS for control, *NC-LPS* microglia were treated with LPS for 24 h, *OE-LPS* microglia were transfected with OE-FANCC before LPS stimulation for 24 h, *KD-LPS* microglia were transfected with KD-FANCC before LPS stimulation for 24 h

To verify the alteration of FANCC levels following SCI, we initially measured the mRNA level of FANCC in injured cords within a week, finding that the expression of FANCC was gradually reduced (Additional file 1: Figure S1A). Additionally, mRNA levels of FANCC in microglia after LPS stimulation for 24 h was significantly decreased compared with controls (Additional file 1: Figure S1B). Furthermore, WB showed a significant decrease of FANCC expression at 7 dpi (Fig. 1A, B). Likewise, the protein levels of FANCC were markedly reduced in microglia after LPS stimulation for 24 h. (Fig. 1C, D). In addition, OE-FANCC and KD-FANCC transfection efficiencies were determined by IF staining, with results indicating that OE increased the expression of FANCC but that KD decreased the expression compared with controls after LPS stimulation (Fig. 1E). The p38-MAPK pathway is a crucial signaling pathway that regulates the inflammatory response in activated microglia [24]. WB indicated that OE-FANCC transfection remarkably reduced the phosphorylated level of p38 in LPS-treated microglia while KD-FANCC increased p38 phosphorylation (Fig. 1F, G). Besides, the level of NLRP3 and ASC, as crucial regulators of inflammation, decreased prominently in OE-FANCC transfected microglia after LPS stimulation, but showed an increase in KD-FANCC transfected microglia (Fig. 1H, I). The results displayed that LPS markedly increased GSDMD/GSDMD-N protein levels, which were reduced by OE-FANCC and increased by KD-FANCC post LPS stimulation (Fig. 1J, K). Additionally, the levels of IL-1β and IL-18 in the cell supernatant were determined, with results indicating that OE-FANCC markedly decreased IL-18 and IL-1β expression, while KD-FANCC enhanced their expression levels after LPS stimulation (Fig. 1L, M). FCA exhibited an increase in the percentage of iNOS positive microglia, OE-FANCC treatment reduced LPS-induced high iNOS positive percentage; however, the silencing of FANCC reduced the iNOS positive percentage in microglia (Fig. 1N, O). However, double staining of TUNEL and IF showed that the reduction of these inflammatory mediators was not due to the death of microglia (Additional file 2: Figure S2A).

### FANCC downregulation of inflammation in microglia is p38/NLRP3 dependent

**Fig. 2:**
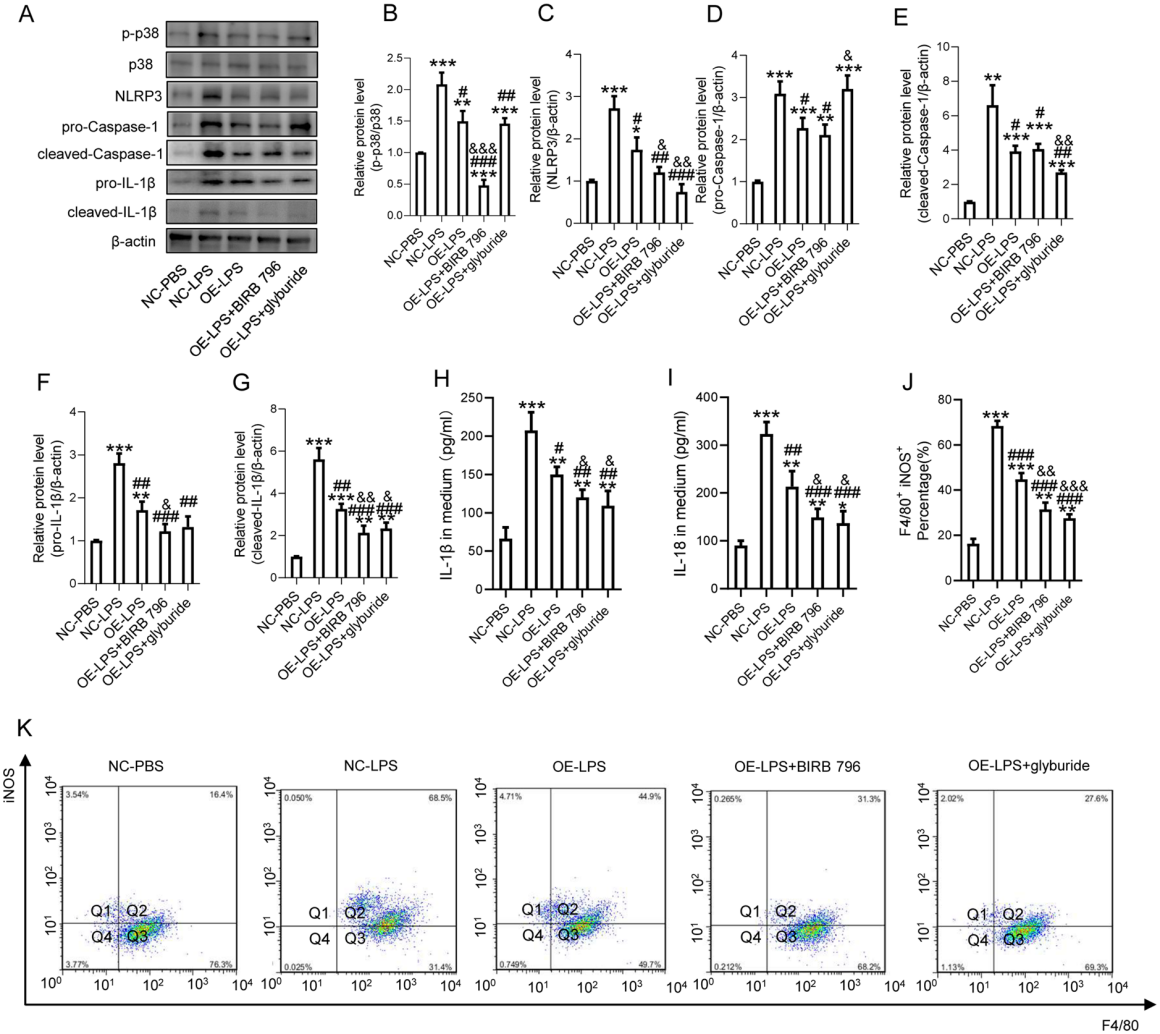
FANCC downregulation of inflammation in microglia is p38/NLRP3 dependent. **A** Western blotting performed for the proteins including p-p38, p38, NLRP3, pro-Caspase-1, cleaved-Caspase-1, pro-IL-1β and cleaved-IL-1β in LPS-activated microglia pretreated with BIRB 796 and glyburide after transfection with OE-FANCC; n = 3. β-actin was used as the control. **B** Bar graph showing the ratio analysis of p-p38/p38. **C** Densitometric analysis of NLRP3 expression. **D** Densitometric analysis of pro-Caspase-1expression. **E** Densitometric analysis of cleaved -Caspase-1expression. **F** Densitometric analysis of pro-IL-1β expression. **G** Densitometric analysis of cleaved -IL-1β expression. **H**, **I** ELISAs performed for the IL-1β and IL-18 in culture medium obtained at 24 h in LPS-activated microglia pretreated with BIRB 796 and glyburide after transfection of OE-FANCC (n = 5). **J** Representative flow cytometry performed for the distinction of iNOS^+^&F4/80^+^ microglia pretreated with BIRB 796 or glyburide and transfected OE-FANCC post 24 h LPS inducement. **K** Bar graph showing the quantitative analysis of the percent; n = 4. The error bars represent the SD. *p < 0.05 vs. NC-PBS group, #p < 0.05 vs. NC-LPS group, &p vs. OE-LPS group by one-way ANOVA followed by Tukey’s post hoc analysis (*p < 0.05, **p < 0.01, and ***p < 0.001)

To further show that FANCC regulated microglial pyroptosis via a p38/NLRP3 dependent pathway, the p38 inhibitor BIRB 796 and an NLRP3 inhibitor glyburide were employed to induce p38/NLRP3 signaling blockade, respectively [25, 26]. WB showed that OE-FANCC decreased p-p38 and NLRP3 protein levels, which were further reduced after treatment with BIRB 796 (Fig. 2A–C). Inversely, KD-FANCC increased the expression of p-p38 and NLRP3, which were remarkedly decreased by treatment with BIRB 796 (Additional file 2: Figure S2B–D); however, NLRP3 inhibition by glyburide did not affect p38 phosphorylation, indicating that FANCC regulated NLRP3 expression via a p38-dependent way during inflammation (Fig. 2A–C). Additionally, OE-FANCC transfection induced a remarkable decrease in the protein levels of pro-caspase-1 and cleaved-caspase-1 in comparation with the LPS-alone treated microglia. However, glyburide increased the expression of pro-caspase-1 and reduced the expression of cleaved-caspase-1 while processing of pro-caspase-1 to the cleaved-caspase-1 was unaffected by BIRB 796 (Fig. 2D, E). Furthermore, the expressions of pro-IL-1β and cleaved-IL-1β were obviously suppressed by the two inhibitors when compared with OE-FANCC transfected microglia (Fig. 2F, G). ELISA showed that both IL-18 and IL-1β expression were further decreased by inhibition of p38 and NLRP3 in OE-FANCC transfected microglia (Fig. 2H, I). FCA revealed that the OE-FANCC-induced decrease in the percentage of F4/80-positive and iNOS-positive cells was markedly reduced by both inhibitors (Fig. 2J, K).

### FANCC reduces microglial inflammation-mediated neuronal apoptosis

**Fig. 3:**
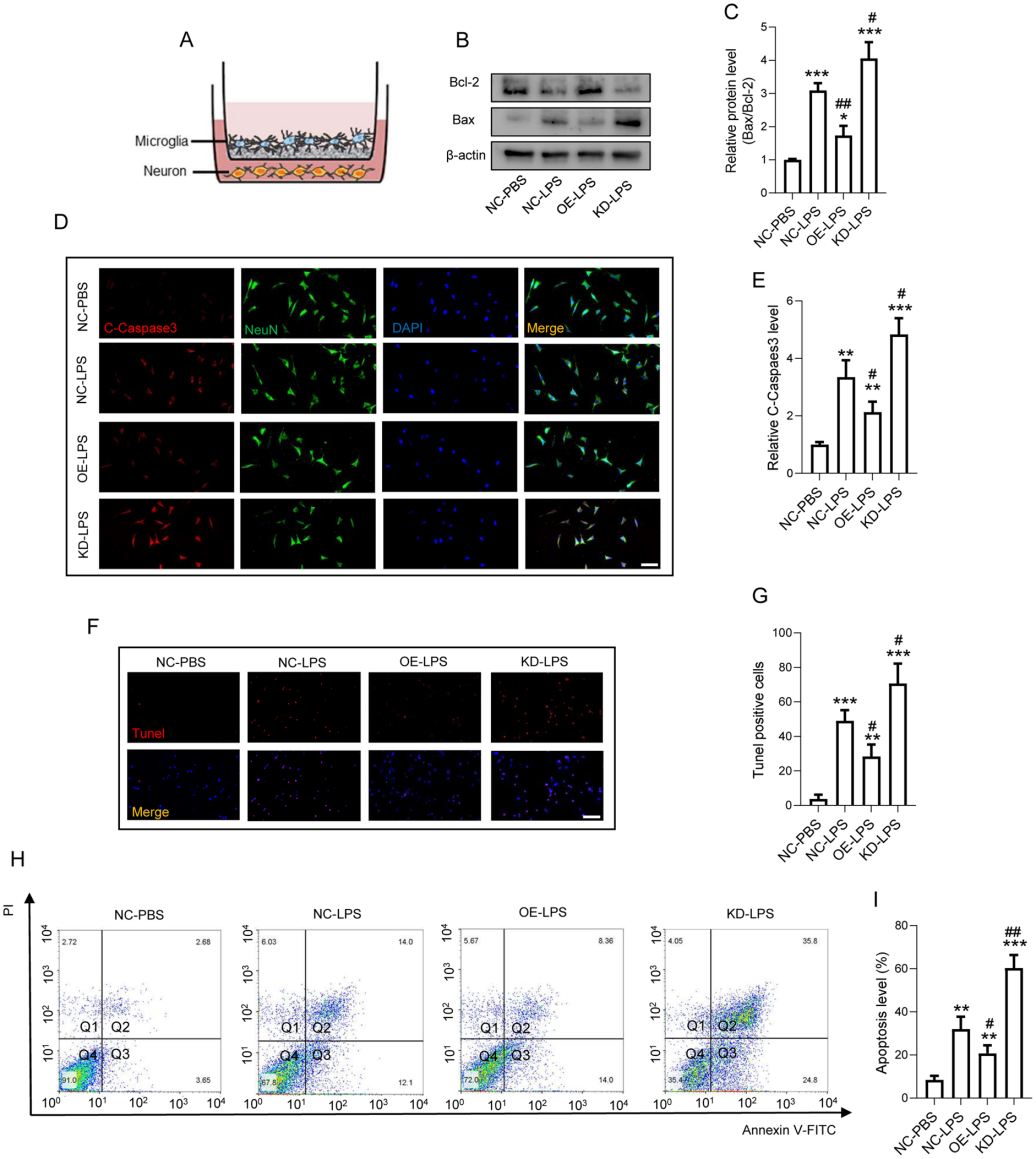
FANCC reduces microglial inflammation-mediated neuronal apoptosis. **A** Diagrammatize method of co-culture between primary microglia and neurons to mimic microglial inflammation-induced neuronal apoptosis in vivo. **B** Western blotting performed for the proteins including Bax and Bcl-2 in primary neurons co-cultured with microglia in NC-PBS, NC-LPS, OE-FANCC-LPS, and KD-FANCC-LPS groups; n = 3. β-actin was used as the control. **C** Bar graph showing the ratio analysis of Bax/Bcl-2. **D** Representative immunofluorescence labeling images for cleaved-Caspase-3 (red) and NeuN (green) obtained from primary neurons co-cultured with microglia in NC-PBS, NC-LPS, OE-LPS, and KD-LPS groups for 48 h, respectively. The blue staining indicates the DAPI-stained nuclei. Scale bar = 50 μm. **E** Quantitative analysis of cleaved-Caspase-3. **F** neuronal apoptosis determined by TUNEL assay in primary neurons co-cultured with microglia. **G** Quantitative analysis of TUNEL-positive cells in primary neurons co-cultured with microglia. **H** Images of FCA with PI and Annexin-V-FITC in primary neurons co-cultured with microglia post LPS treatment for 24 h.** I** Quantitative analysis of the apoptosis level of neurons; n = 4. The error bars represent the SD. *p < 0.05 vs. NC-PBS group, #p < 0.05 vs. NC-LPS group by one-way ANOVA followed by Tukey's post hoc analysis (*p < 0.05, **p < 0.01, and ***p < 0.001)

As shown in Fig. 3A, neurons and microglia were co-cultured in cell inserts to mimic microglial inflammation-induced neuronal apoptosis in vivo. WB showed that the ratio of Bax/Bcl-2 increased in neurons cultured with LPS-treated microglia, which was significantly reduced in the OE-FANCC group. However, co-culture with KD-FANCC transfected microglia remarkably increased the ratio of Bax/Bcl-2 compared with that in the NC-LPS group (Fig. 3B, C). Moreover, IF staining showed an increased level of cleaved-caspase3 in neurons after microglia activation; however, such and increase was markedly suppressed by OE-FANCC transfection but was observably increased by KD-FANCC transfection (Fig. 3D, E). Furthermore, TUNEL assay results indicated that the activated microglia transfected with OE-FANCC had significantly reduced numbers of TUNEL-positive neurons compared with the NC-LPS group, while KD-FANCC transfection led to a prominent increase of apoptotic neurons post LPS stimulation (Fig. 3F, G). FCA further showed that the percentage of apoptotic neurons significantly decreased in the OE-FANCC group but was increased after KD-FANCC transfection compared with LPS-alone treated microglia (Fig. 3H, I).

### Silencing FANCC exacerbates SCI-induced pyroptosis and NLRP3-dependent neuroinflammation

**Fig. 4:**
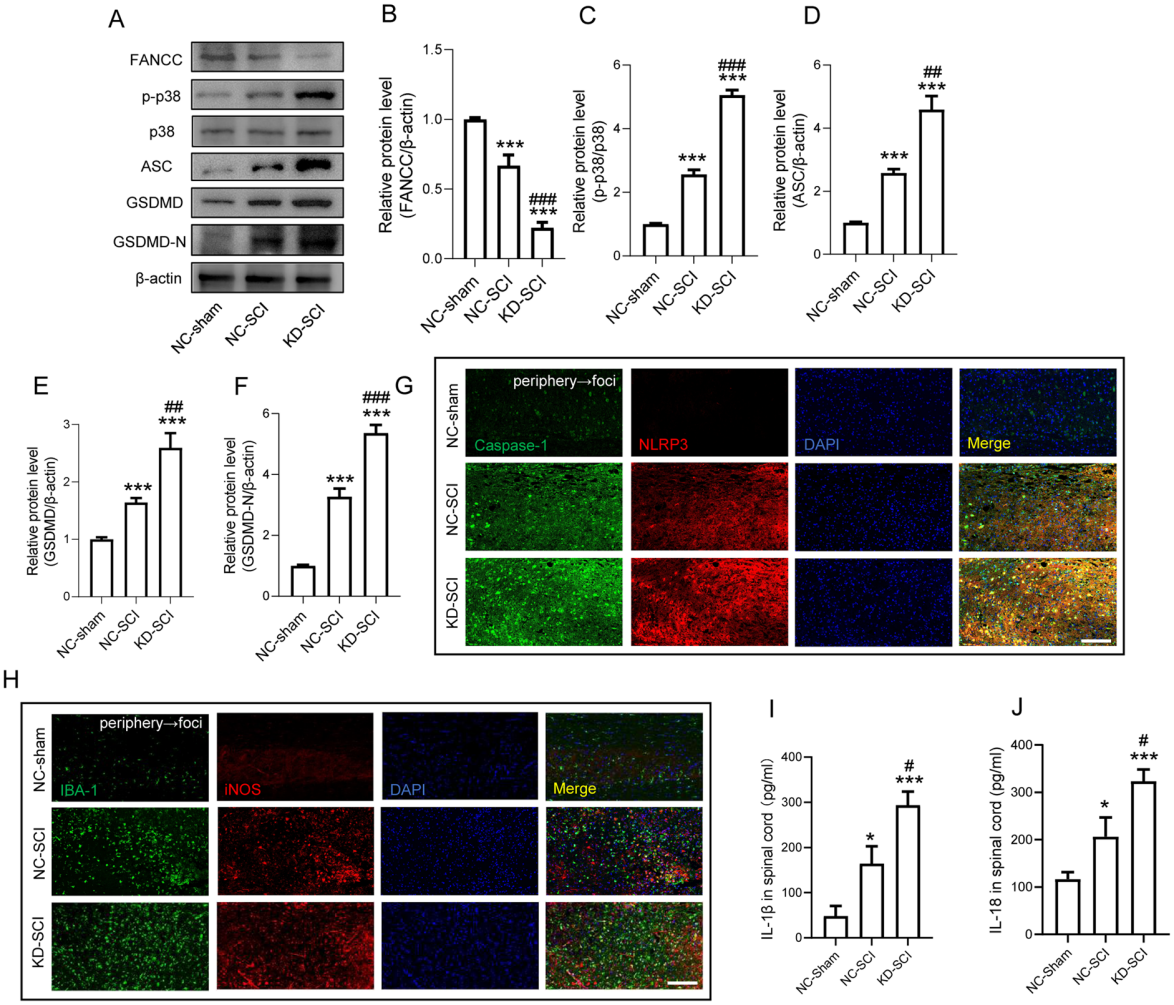
Silencing FANCC exacerbates SCI-induced pyroptosis and NLRP3-depended neuroinflammation. **A** Western blotting of FANCC, p-p38, p38, ASC, GSDMD and GSDMD-N protein levels at 3 dpi in NC-sham, NC-SCI and KD-SCI mice. **B** Bar graph showing a quantitative analysis of FANCC expression; n = 3. **C** Bar graph showing the ratio of p-p38/p38; n = 3. **D** Densitometric analysis of ASC expression. **E** Densitometric analysis of GSDMD expression. **F** Densitometric analysis of GSDMD-N expression. **G** Double immunofluorescence of Caspase-1 (green) and NLRP3 (red), obtained from longitudinal sections centered around central canal at 3 dpi in Sham, SCI and KD-SCI mice. Scale bar = 100 μm. **H** Double immunofluorescence labeling of microglia for IBA-1(green) and iNOS (red), obtained from longitudinal Sects. 1 mm caudal to the lesion site at 3 dpi in Sham, SCI and KD-SCI mice. As shown in the KD-SCI mice, iNOS was increased in the microglia. Scale bar = 100 μm. **I**, **J** ELISAs performed for the IL-1β and IL-18 expressions in injured tissue obtained at 3 dpi, showing significantly increased levels of IL-1β and IL-18 in KD-SCI group; n = 5. The error bars represent the SD. *p < 0.05 vs. NC-sham group, #p < 0.05 vs. NC-SCI group by one-way ANOVA followed by Tukey’s post hoc analysis (*p < 0.05, **p < 0.01, and ***p < 0.001). *NC-sham* mice were performed laminectomy only, *NC-SCI* mice were performed spinal cord contusion, *KD-SCI* mice were transfected with KD-FANCC before SCI

We established the SCI model in KD-FANCC mice and attempted to clarify the role of FANCC in pyroptosis during post-SCI neuroinflammation. At 3 dpi, FANCC protein levels were decreased in SCI mice and markedly reduced in KD-SCI mice (Fig. 4A, B). Phosphorylated levels of p38 were increased after SCI, which was further exacerbated in KD-FANCC mouse spinal cords (Fig. 4C). Moreover, the expressions of the pyroptosis-related proteins including ASC, GSDMD and GSDMD-N increased following SCI, which were prominently increased in KD-SCI mice (Fig. 4D–F). Additionally, IF staining showed that the fluorescence intensity of both caspase-1 and NLRP3 was increased after SCI, and became more evident in KD-SCI mice (Fig. 4G). At 3 dpi that KD-SCI mice had increased numbers of inflammatory microglia in comparison with that in the NC-SCI group (Fig. 4H). Importantly, the neuroinflammatory response in the injured cords at 3 dpi, as detected by the NLRP3-induced inflammatory cytokines IL-1β and IL-18, was significantly increased in SCI mice in comparison with sham-treated mice. As expected, KD-SCI mice had higher levels of these inflammatory factors post SCI (Fig. 4I, J).

### FANCC deficiency aggravates glial scar formation, myelin sheath destruction and axon outgrowth impairment

**Fig. 5:**
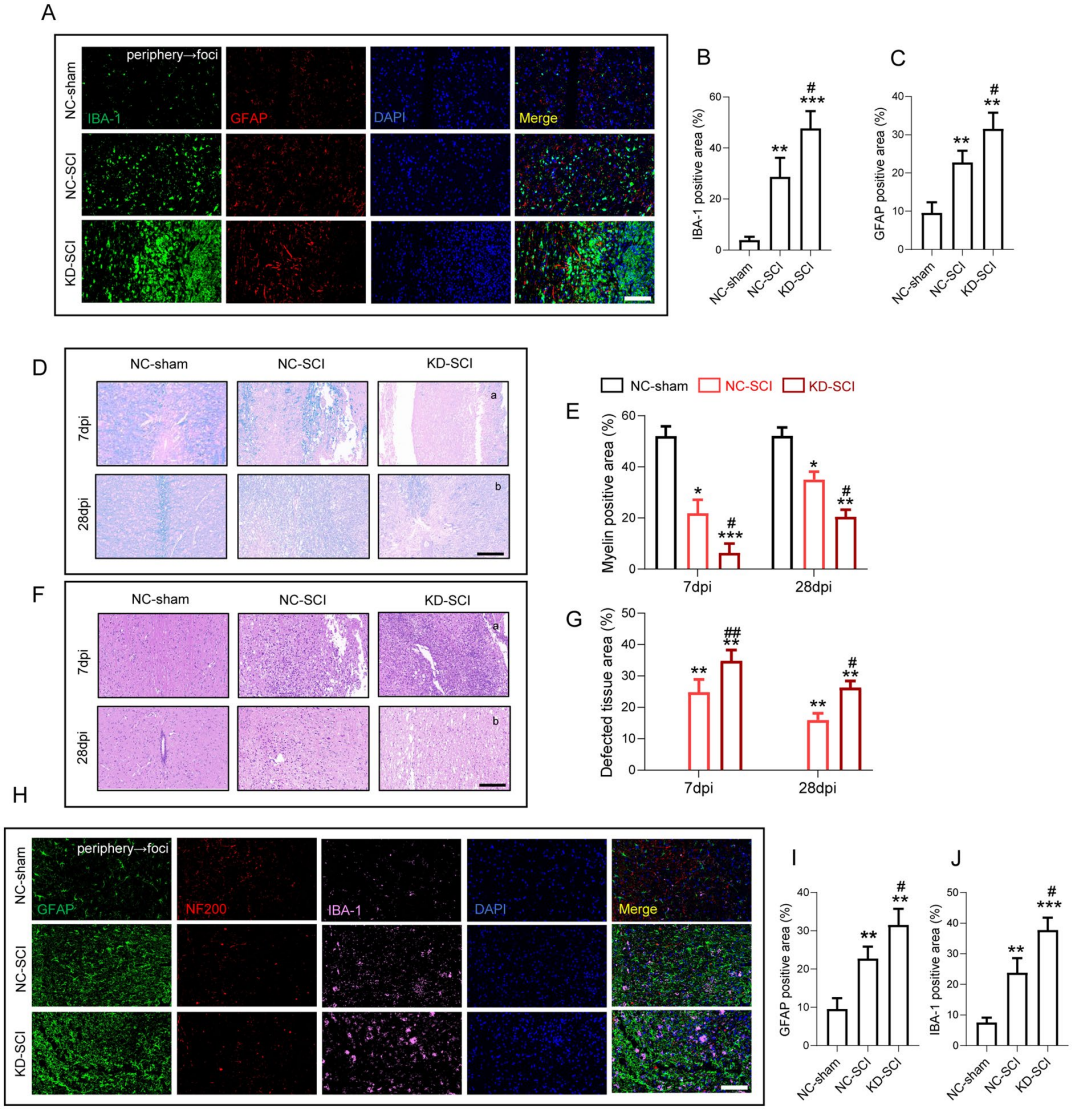
FANCC deficiency aggravates glial scar formation, myelin sheath destruction and axon outgrowth impairment. **A** Double immunofluorescence labeling of microglia for IBA-1 (green) and astrocytes for GFAP (red) obtained from longitudinal sections centered around the injured core 3 mm at 7 dpi in NC-sham, NC-SCI and KD-SCI mice; Scale bar = 500 μm. **B** Quantitative analysis of the area of microglia scar at 7dpi; n = 6. **C** Quantitative analysis of the area of astrocyte scar at 7dpi; n = 6. **D** Representative images for LFB staining obtained from longitudinal sections centered around the injured core 3 mm at **a** 7 and **b** 28 dpi in NC-sham, NC-SCI and KD-SCI mice; Scale bar = 500 μm. **E** Quantitative analysis of the demyelinated area at 7and 28 dpi; n = 6.** F** HE staining images of cords centered around the injured core 3 mm obtained at (a) 7 and (b) 28 dpi; Scale bar = 500 μm. **G** Quantitative analysis of the defected area at 7and 28 dpi; n = 6. **H** Triple immunofluorescence labeling of microglia for IBA-1 (pink), astrocytes for GFAP (green) and neurons and neurofilaments for NF-200 (red) obtained from longitudinal sections centered around the injured core 3 mm at 28 dpi in NC-sham, NC-SCI and KD-SCI mice; Scale bar = 500 μm. **I** Quantitative analysis of the area of astrocyte scar at 28 dpi; n = 6. **J** Quantitative analysis of the area of microglia scar at 28 dpi; n = 6. The error bars represent the SD. *p < 0.05 vs. NC-sham group, #p < 0.05 vs. NC-SCI group by one-way ANOVA followed by Tukey’s post hoc analysis (*p < 0.05, **p < 0.01, and ***p < 0.001)

To accurately analyze the glial cell area at 7 dpi, we selected a 2 mm length of tissue, which was close to the lesion epicenter for further IF analysis. The results indicated that both the IBA-1- and GFAP-positive area was increased following SCI. In agreement with WB and ELISA results, KD-SCI mice showed a larger GFAP-positive area and an increased IBA-1-positive area as compared with NC-SCI mice (Fig. 5A–C). In addition, LFB staining at 7 and 28 dpi showed that compared with the NC-sham group, SCI resulted in extensive areas of demyelination, which were exacerbated in KD-SCI mice in spite of a compensatory myelin repair after SCI (Fig. 5D, E). Besides, the neurohistological integrity of the injured cords at 7 and 28 dpi, as visualized by HE staining, indicated greater cell infiltration at 7 dpi and a more serious tissue destruction of cords at 28 dpi in KD-SCI mice, compared with the NC-sham and NC-SCI groups (Fig. 5F, G). At 28 dpi, triple IF staining revealed that KD-SCI mice had significantly increased expression of IBA-1 and GFAP, and decreased expression of NF-200 compared with compared with NC-SCI mice (Fig. 5H − J).

### Knockdown of FANCC interferes with locomotor recovery by increasing neuronal apoptosis in SCI mice

**Fig. 6:**
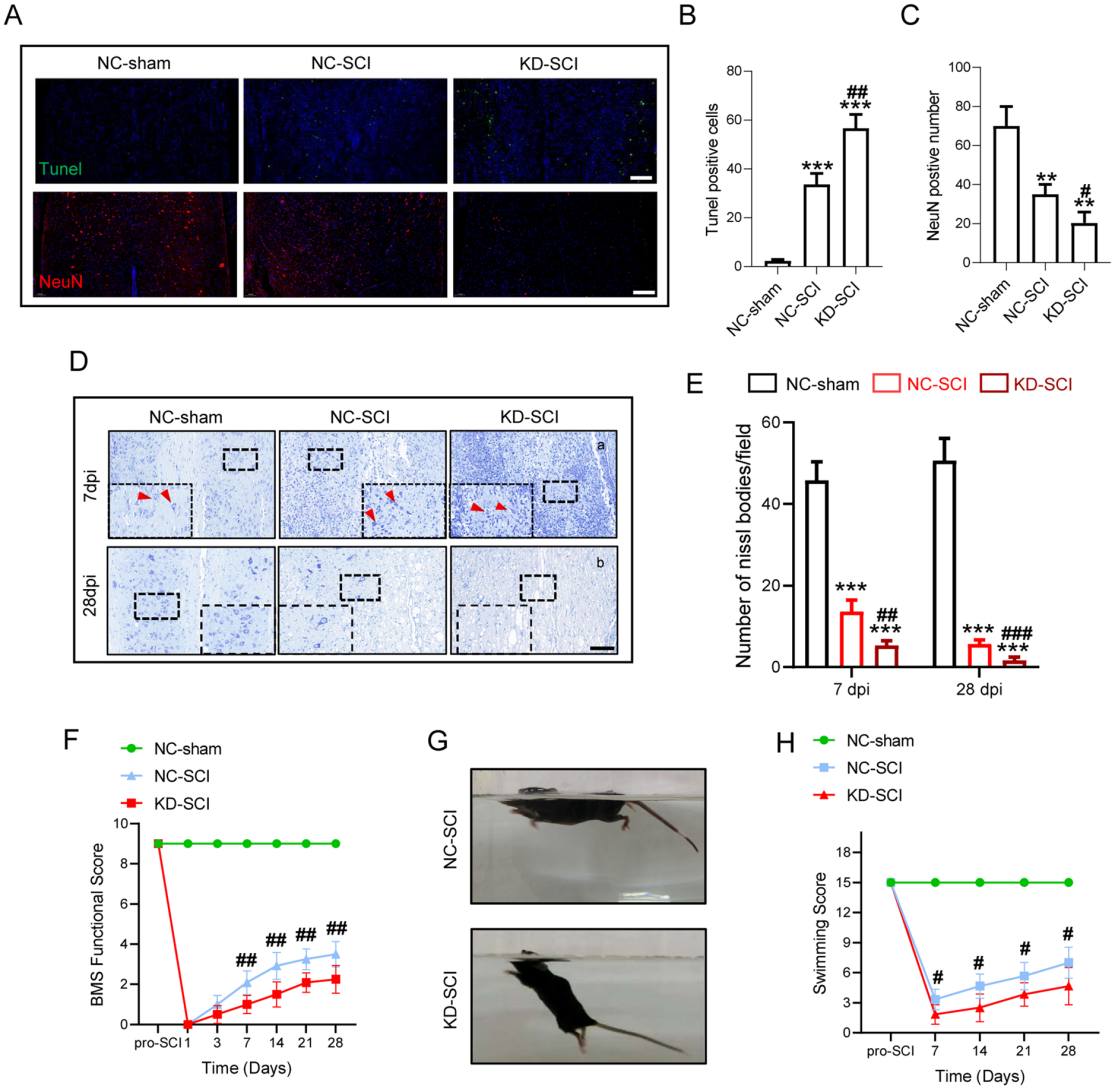
Knockdown of FANCC interferes with locomotor recovery by increasing neuronal apoptosis in SCI mice. **A** tissular apoptosis and neuronal loss were determined by TUNEL assay and IF at 7 dpi in NC-sham, NC-SCI and KD-SCI mice. **B** Quantitative analysis of TUNEL-positive cells. **C** Quantitative analysis of NeuN-positive neurons. **D** Representative images for Nissl staining obtained from longitudinal sections centered around the injured core 1.5 mm at **a** 7 dpi and **b** 28 dpi in NC-sham, NC-SCI and KD-SCI mice. Scale bar = 250 μm.** E** Quantitative analysis of the amounts of survived neurons at 7 and 28 dpi; n = 6. **F** The BMS score post SCI in NC-sham, NC-SCI and KD-SCI mice. **G**, **H** Photos of Swimming at 28 dpi, showing the worse trunk instability and uncoordinated action in SCI mice, and statistical analysis of the LSS over a 28 days period; n = 6. *p < 0.05 vs. NC-sham group, #p < 0.05 vs. NC-SCI group by one-way ANOVA followed by Tukey’s post hoc analysis (*p < 0.05, **p < 0.01, and ***p < 0.001)

To confirm the function of FANCC in neuronal apoptosis post SCI, TUNEL staining was used to measure the severity of apoptotic cords, with results indicating that the amount of TUNEL-positive tissue was significantly increased in KD-SCI mice compared with NC-SCI mice at 7 dpi. IF staining revealed that KD-SCI mice had significantly increased the loss of neurons (Fig. 6A–C). Moreover, Nissl staining was performed at 7 and 28 dpi, with results indicating that the number of neurons in KD-SCI mice was decreased compared with that in NC-SCI and NC-sham mice (Fig. 6D, E). Importantly, the nucleus and membrane structure were blurred in neurons of NC-SCI mice, which was markedly aggravated in KD-SCI mice at 7 dpi, which further indicated that FANCC deficiency affected the degree of neuronal apoptosis (Fig. 6D, E). For locomotor functional assessment, the BMS and LSS  were employed to determine the locomotor rehabilitation of SCI mice. The BMS scores in the NC-sham group were in the normal range after laminectomy, but mice in the other two groups showed low scores at 3 dpi. Notably, the scores of KD-SCI mice were significantly less than SCI mice starting on 7 dpi and lasting until 28 dpi (Fig. 6F). The LSS also indicated that KD-FANCC SCI mice presented with poorer body balance, more forelimb dependence, weaker hindlimb alternations and a larger body-surface angle starting at 7 dpi (Fig. 6G, H). Taken together, the results indicate that reduced FANCC expression caused an increase in neuronal apoptosis and thus impaired locomotor recovery post SCI.

## Discussion

The secondary phase of SCI is initiated by a series of pathophysiological events that are initiated by a primary mechanical contusion in the spinal cord [27–29]. Aberrant neuroinflammation during SCI is activated after mechanical trauma, and, as such, suppression of neuroinflammation is supposed to be a therapeutic method to prevent the deterioration of SCI [30, 31]. The activation of p38 and the NLRP3 inflammasome is involved in the initiation of neuroinflammation post SCI [32, 33]. Furthermore, NLRP3 is also reported to mediate pyroptosis in other CNS diseases, such as experimental autoimmune encephalomyelitis and neuromyelitis [34, 35]. However, the genesis and progression of microglial pyroptosis in SCI remains uncertain. On this basis, our research focused on the biological role of FANCC in microglial pyroptosis and subsequent neuronal apoptosis via its regulation of the p38/NLRP3 axis post SCI.

FANCC is a member of the FA gene family, which has been mainly studied in FA and cancers [36–38]. Additionally, FANCC is involved in inflammatory response in disease models. In particular, Daniel et al. reported an intensive inflammatory response to LPS-induced septic shock in FANCC-deficient mice [39]. Nevertheless, its pathophysiological function in inflammatory events has not been extensively researched in CNS injury and, in particular, in SCI. Our findings showed that as the level of FANCC decreased the activation of p38 and NLRP3 in injured spinal cords and LPS-treated microglia increased. Interestingly, further overexpression of FANCC markedly reduced the phosphorylation of p38 and reduced NLRP3 expression along with several inflammatory cytokines in activated microglia, while knockdown of FANCC reversed these outcomes. In the initiation of pyroptosis, the NLRP3 inflammasome, containing ASC and pro-caspase1, promotes activation of cleaved-caspase-1, which cleaves GSDMD, and ultimately leads to generation of IL-1β and IL-18 [40, 41]. Here, the levels of pyroptosis-related proteins such as GSDMD/GSDMD-N and ASC were similar to those of p38 and NLRP3 in OE/KD-FANCC treated microglia. In parallel, in vivo results indicated that KD-FANCC SCI mice showed higher levels of p38 and NLRP3 as well as the pyroptosis markers GSDMD/GSDMD-N and ASC. The results suggested an internal mechanism by which FANCC knockdown promoted pyroptosis and neuroinflammation caused by microglia via the p38/NLRP3 pathway. As previously mentioned, the NLRP3 inflammasome is a crucial regulator associated with pyroptosis and various inflammatory responses. NLRP3 facilitates the release of various inflammatory factors including IL-1β and IL-18, which are prominent amplifiers of neuroinflammation [42, 43]. Here, we showed that p38 was an upstream target of NLRP3, which was regulated by FANCC to inhibit microglial pyroptosis and neuroinflammation. Furthermore, application of the p38 inhibitor BIRB 796 markedly decreased NLRP3 levels but application of the NLRP3 inhibitor glyburide did not affect p38 expression after OE-FANCC transfection. Furthermore, OE-FANCC transfection along with inhibition of p38 and NLRP3 markedly reduced IL-1β and IL-18 expression and attenuated neuroinflammation.

Neuronal apoptosis, which is a form of programmed cell death that occurs in neurons, has long been regarded as a dominant cause motor deficiency in a number of neurological diseases including SCI [44–46]. The severity of neuronal apoptosis can lead to irreversible damage to nervous tissue followed by demyelination and motor system degeneration post SCI [47, 48]. Several studies have reported that activated microglia produce abundant inflammatory mediators such as IL-1, nitric oxide, tumor necrosis factor alpha, prostaglandin E2 and superoxide, which are toxic to neurons and result in neuronal apoptosis. [49, 50]. In our study, microglial inflammation-induced neuronal apoptosis was simulated by co-culture of neurons and activated microglia in vitro. OE-FANCC transfection decreased the ratio of Bax/Bcl-2 and reduced the levels of caspase-3 and Annexin-V, whereas transfection with KD-FANCC had the opposite effects. Likewise, TUNEL-positive neurons were significantly decreased after OD-FANCC transfection but were increased after KD-FANCC transfection both in vitro and in vivo. Furthermore, the number of surviving neurons in KD-SCI mice was decreased compared with SCI mice. These results indicate that FANCC deficiency promotes neuronal apoptosis caused by microglial-induced neuroinflammation.

Glial scar formation, myelin sheath destruction and axon outgrowth impediment are common histological conditions post SCI, which ultimately result in neurological dysfunction [51, 52]. Our findings indicated that KD-FANCC SCI mice showed more damages to the myelin sheath and cord tissue in comparison with SCI mice. In addition, neuroinflammation post SCI is known to be characterized by an increased area of activated microglia and astrocytes, which are IBA-1- and GFAP-positive, respectively [53]. Compared with the SCI mice, KD-FANCC mice had an increased glial scar both at 7 and 28 dpi. Furthermore, axon outgrowth was more severely inhibited in KD-FANCC mice compared with SCI mice. Previous studies have shown that neurological damage post SCI often causes perception disabilities, such as sensory dysfunction and paralysis [54]. Our results indicated that FANCC deficiency may lead to more severe damage to nervous tissue post SCI.

**Fig. 7:**
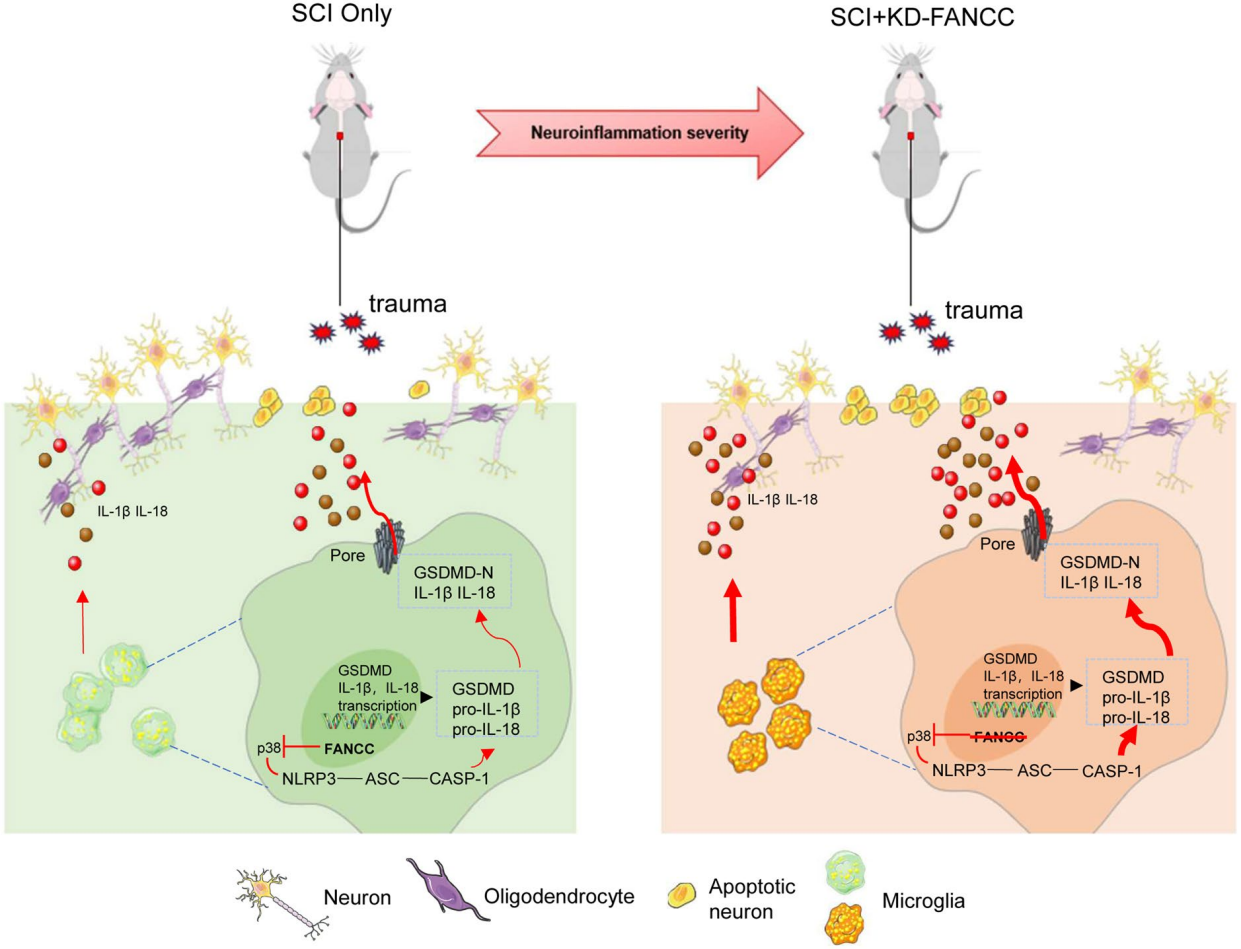
Pyroptosis mediated by the pore-forming protein GSDMD is a necessary step of neuroinflammation and subsequent neuronal apoptosis after SCI. We demonstrated that deficiency of FANCC, a member of the FA gene family, can aggravate microglial pyroptosis and neuronal apoptosis via p38/NLRP3 signaling pathway

Here, we initially confirmed that increased expression of FANCC in SCI mice and LPS-stimulated microglia markedly inhibited pyroptosis and neuroinflammation via blocking the p38/NLRP3 pathway. In addition, KD-FANCC transfection in SCI mice and neurons co-cultured with microglia aggravated neuronal apoptosis both in vitro and in vivo, ultimately resulting in degeneration of neural tissue and locomotor function. These findings clarified the mechanism of FANCC in attenuating pyroptosis and neuronal apoptosis following SCI (Fig. 7). However, the specific regulatory factors or targets involved in mediating pyroptosis and neuronal apoptosis after SCI remain uncertain and whether there is an upstream regulator that affects the expression of FANCC is still unknown. Thus, in-depth studies of FANCC in SCI needed to be implemented in the future.

## Supplementary Information

**Additional file 1: Figure S1.** A Relative mRNA level of FANCC in the spinal cord within a week post-injury; n = 5. The error bars represent the SD. *p < 0.05 vs. Sham group by one-way ANOVA followed by Tukey’s post hoc analysis (*p < 0.05, **p < 0.01, and ***p < 0.001). B Relative mRNA level of FANCC in LPS-stimulated primary microglia for 12h; n = 5. The error bars represent the SD. *p < 0.05 vs. PBS group by t-test (*p < 0.05, **p < 0.01, and ***p < 0.001).

**Additional file 2: Figure S2.** A Microglial death determined by TUNEL assay in LPS-activated microglia after transfection with OE-FANCC and KD-FANCC; Scale bar = 200 μm. B Western blotting performed for p-p38, p38 and NLRP3 in LPS-activated microglia pretreated with BIRB 796 after transfection with KD-FANCC; n = 3. GAPDH was used as the control. C Bar graph showing the ratio analysis of p-p38/p38. D Densitometric analysis of NLRP3 expression. The error bars represent the SD. *p < 0.05 vs. NC-PBS group, #p < 0.05 vs. NC-LPS group, &p vs. KD-LPS group by one-way ANOVA followed by Tukey's post hoc analysis (*p < 0.05, **p < 0.01, and ***p < 0.001).

## Data Availability

All data generated or analysed during this study are included in this published article and its supplementary information files.

## Abbreviations

AAV: Adeno-associated virus
ASC: Apoptosis-associated speck-like protein containing CARD
BMS: Basso mouse scale
CNS: Central nervous system
DAPI: Diaminobenzidine
DMEM: Dulbecco’s modified Eagle medium
dpi: Days post injury
ELISAs: Enzyme-linked immunosorbent assays
FANCC: Fanconi anemia group C complementation group gene
FBS: Fetal bovine serum
FCA: Flow cytometry assay
GSDMD-N: N-terminus pore-forming protein gasdermin D
HE: Hematoxylin–eosin
IF: Immunofluorescence
IL-1β: Interleukin-1β
KD: Knockdown
LFB: Luxol fast blue
LPS: Lipopolysaccharide
NLR: Nod-like receptor
OE: Overexpression
SCI: Spinal cord injury
sh: Short hairpin
WB: Western blotting

## References

[1] Silva NA, Sousa N, Reis RL, Salgado AJ (2014). From basics to clinical: a comprehensive review on spinal cord injury. Prog Neurobiol.

[2] McDonald JW, Sadowsky C (2002). Spinal-cord injury. Lancet.

[3] Saadoun S, Papadopoulos MC (2020). Targeted perfusion therapy in spinal cord trauma. Neurotherapeutics.

[4] Wang C, Wang Q, Lou Y, Xu J, Feng Z, Chen Y, Tang Q, Zheng G, Zhang Z, Wu Y, Tian N, Zhou Y, Xu H, Zhang X (2018). Salidroside attenuates neuroinflammation and improves functional recovery after spinal cord injury through microglia polarization regulation. J Cell Mol Med.

[5] Chen J, Wang Z, Zheng Z, Chen Y, Khor S, Shi K, He Z, Wang Q, Zhao Y, Zhang H, Li X, Li J, Yin J, Wang X, Xiao J (2017). Neuron and microglia/macrophage-derived FGF10 activate neuronal FGFR2/PI3K/Akt signaling and inhibit microglia/macrophages TLR4/NF-κB-dependent neuroinflammation to improve functional recovery after spinal cord injury. Cell Death Dis.

[6] Hutson TH, Di Giovanni S (2019). The translational landscape in spinal cord injury: focus on neuroplasticity and regeneration. Nat Rev Neurol.

[7] Fan H, Zhang K, Shan L, Kuang F, Chen K, Zhu K, Ma H, Ju G, Wang Y (2016). Reactive astrocytes undergo M1 microglia/macrohpages-induced necroptosis in spinal cord injury. Mol Neurodegener.

[8] Kesavardhana S, Malireddi R, Kanneganti TD (2020). Caspases in cell death, inflammation, and pyroptosis. Annu Rev Immunol.

[9] Frank D, Vince JE (2019). Pyroptosis versus necroptosis: similarities, differences, and crosstalk. Cell Death Differ.

[10] Tsuchiya K (2021). Switching from apoptosis to pyroptosis: gasdermin-elicited inflammation and antitumor immunity. Int J Mol Sci.

[11] Al Mamun A, Wu Y, Monalisa I, Jia C, Zhou K, Munir F, Xiao J (2020). Role of pyroptosis in spinal cord injury and its therapeutic implications. J Adv Res.

[12] Kovacs SB, Miao EA (2017). Gasdermins: effectors of pyroptosis. Trends Cell Biol.

[13] Shi J, Zhao Y, Wang K, Shi X, Wang Y, Huang H, Zhuang Y, Cai T, Wang F, Shao F (2015). Cleavage of GSDMD by inflammatory caspases determines pyroptotic cell death. Nature.

[14] Liu Z, Yao X, Jiang W, Li W, Zhu S, Liao C, Zou L, Ding R, Chen J (2020). Advanced oxidation protein products induce microglia-mediated neuroinflammation via MAPKs-NF-κB signaling pathway and pyroptosis after secondary spinal cord injury. J Neuroinflammation.

[15] McKenzie BA, Dixit VM, Power C (2020). Fiery cell death: pyroptosis in the central nervous system. Trends Neurosci.

[16] Liu Y, Ballman K, Li D, Khan S, Derr-Yellin E, Shou W, Haneline LS (2012). Impaired function of Fanconi anemia type C-deficient macrophages. J Leukoc Biol.

[17] Heinrich MC, Silvey KV, Stone S, Zigler AJ, Griffith DJ, Montalto M, Chai L, Zhi Y, Hoatlin ME (2000). Posttranscriptional cell cycle-dependent regulation of human FANCC expression. Blood.

[18] Chandrasekharappa SC, Lach FP, Kimble DC, Kamat A, Teer JK, Donovan FX, Flynn E, Sen SK, Thongthip S, Sanborn E, Smogorzewska A, Auerbach AD, Ostrander EA (2013). Massively parallel sequencing, aCGH, and RNA-Seq technologies provide a comprehensive molecular diagnosis of Fanconi anemia. Blood.

[19] Zha J, Kunselman L, Fan JM, Olson TS (2019). Bone marrow niches of germline FANCC/FANCG deficient mice enable efficient and durable engraftment of hematopoietic stem cells after transplantation. Haematologica.

[20] Hadjur S, Jirik FR (2003). Increased sensitivity of Fancc-deficient hematopoietic cells to nitric oxide and evidence that this species mediates growth inhibition by cytokines. Blood.

[21] Qian Z, Chang J, Jiang F, Ge D, Yang L, Li Y, Chen H, Cao X (2020). Excess administration of miR-340-5p ameliorates spinal cord injury-induced neuroinflammation and apoptosis by modulating the P38-MAPK signaling pathway. Brain Behav Immun.

[22] Basso DM, Fisher LC, Anderson AJ, Jakeman LB, McTigue DM, Popovich PG (2006). Basso Mouse Scale for locomotion detects differences in recovery after spinal cord injury in five common mouse strains. J Neurotrauma.

[23] Smith RR, Burke DA, Baldini AD, Shum-Siu A, Baltzley R, Bunger M, Magnuson DSK (2006). The Louisville Swim Scale: a novel assessment of hindlimb function following spinal cord injury in adult rats. J Neurotrauma.

[24] Song C, Heping H, Shen Y, Jin S, Li D, Zhang A, Ren X, Wang K, Zhang L, Wang J, Shi D (2020). AMPK/p38/Nrf2 activation as a protective feedback to restrain oxidative stress and inflammation in microglia stimulated with sodium fluoride. Chemosphere.

[25] Rohm S, Schroder M, Dwyer JE, Widdowson CS, Chaikuad A, Berger BT, Joerger AC, Kramer A, Harbig J, Dauch D, Kudolo M, Laufer S, Bagley MC, Knapp S (2020). Selective targeting of the alphaC and DFG-out pocket in p38 MAPK. Eur J Med Chem.

[26] Lamkanfi M, Mueller JL, Vitari AC, Misaghi S, Fedorova A, Deshayes K, Lee WP, Hoffman HM, Dixit VM (2009). Glyburide inhibits the Cryopyrin/Nalp3 inflammasome. J Cell Biol.

[27] Ahuja CS, Wilson JR, Nori S, Kotter MRN, Druschel C, Curt A, Fehlings MG (2017). Traumatic spinal cord injury. Nat Rev Dis.

[28] Dias DO, Kim H, Holl D, Werne Solnestam B, Lundeberg J, Carlén M, Göritz C, Frisén J (2018). Reducing pericyte-derived scarring promotes recovery after spinal cord injury. Cell.

[29] Pelisch N, Rosas Almanza J, Stehlik KE, Aperi BV, Kroner A (2020). CCL3 contributes to secondary damage after spinal cord injury. J Neuroinflammation.

[30] Zeng H, Liu N, Yang Y, Xing H, Liu X, Li F, La G, Huang M, Zhou M (2019). Lentivirus-mediated downregulation of α-synuclein reduces neuroinflammation and promotes functional recovery in rats with spinal cord injury. J Neuroinflammation.

[31] Rong Y, Liu W, Wang J, Fan J, Luo Y, Li L, Kong F, Chen J, Tang P, Cai W (2019). Neural stem cell-derived small extracellular vesicles attenuate apoptosis and neuroinflammation after traumatic spinal cord injury by activating autophagy. Cell Death Dis.

[32] Wang J, Ren C, Feng J, Ou C, Liu L (2020). Oleanolic acid inhibits mouse spinal cord injury through suppressing inflammation and apoptosis via the blockage of p38 and JNK MAPKs. Biomed Pharmacother.

[33] Jiang W, Li M, He F, Zhou S, Zhu L (2017). Targeting the NLRP3 inflammasome to attenuate spinal cord injury in mice. J Neuroinflammation.

[34] Li S, Wu Y, Yang D, Wu C, Ma C, Liu X, Moynagh PN, Wang B, Hu G, Yang S (2019). Gasdermin D in peripheral myeloid cells drives neuroinflammation in experimental autoimmune encephalomyelitis. J Exp Med.

[35] Peng Y, Chen J, Dai Y, Jiang Y, Qiu W, Gu Y, Wang H (2019). NLRP3 level in cerebrospinal fluid of patients with neuromyelitis optica spectrum disorders: Increased levels and association with disease severity. Mult Scler Relat Disord.

[36] Pulliam-Leath AC, Ciccone SL, Nalepa G, Li X, Si Y, Miravalle L, Smith D, Yuan J, Li J, Anur P, Orazi A, Vance GH, Yang F, Hanenberg H, Bagby GC, Clapp DW (2010). Genetic disruption of both Fancc and Fancg in mice recapitulates the hematopoietic manifestations of Fanconi anemia. Blood.

[37] Pan Z, Wang X, Chen T, Ding X, Jiang X, Gao Y, Mo W, Huang Y, Lou C, Cao W (2019). Deleterious mutations in DNA repair gene FANCC exist in BRCA1/2-Negative Chinese familial breast and/or Ovarian Cancer Patients. Front Oncol.

[38] Berger G, van den Berg E, Smetsers S, Leegte BK, Sijmons RH, Abbott KM, Mulder AB, Vellenga E (2019). Fanconi anaemia presenting as acute myeloid leukaemia and myelodysplastic syndrome in adulthood: a family report on co-occurring FANCC and CHEK2 mutations. Br J Haematol.

[39] Sejas DP, Rani R, Qiu Y, Zhang X, Fagerlie SR, Nakano H, Williams DA, Pang Q (2007). Inflammatory reactive oxygen species-mediated hematopoietic suppression in Fancc-deficient mice. J Immunol.

[40] Li N, Zhou H, Wu H, Wu Q, Duan M, Deng W, Tang Q (2019). STING-IRF3 contributes to lipopolysaccharide-induced cardiac dysfunction, inflammation, apoptosis and pyroptosis by activating NLRP3. Redox Biol.

[41] He Y, Hara H, Nunez G (2016). Mechanism and regulation of NLRP3 inflammasome activation. Trends Biochem Sci.

[42] Chen Y, Meng J, Bi F, Li H, Chang C, Ji C, Liu W (2019). EK7 regulates NLRP3 inflammasome activation and neuroinflammation post-traumatic brain injury. Front Mol Neurosci.

[43] Xu S, Wang J, Zhong J, Shao M, Jiang J, Song J, Zhu W, Zhang F, Xu H, Xu G, Zhang Y, Ma X, Lyu F (2021). CD73 alleviates GSDMD-mediated microglia pyroptosis in spinal cord injury through PI3K/AKT/Foxo1 signaling. Clin Transl Med.

[44] Luo X, Li L, Xu W, Cheng Y, Xie Z (2020). HLY78 attenuates neuronal apoptosis via the LRP6/GSK3β/β-catenin signaling pathway after subarachnoid hemorrhage in rats. Neurosci Bull.

[45] Gu C, Li L, Huang Y, Qian D, Liu W, Zhang C, Luo Y, Zhou Z, Kong F, Zhao X, Liu H, Gao P, Chen J, Yin G (2020). Salidroside ameliorates mitochondria-dependent neuronal apoptosis after spinal cord ischemia-reperfusion injury partially through inhibiting oxidative stress and promoting mitophagy. Oxid Med Cell Longev.

[46] Tran AP, Warren PM, Silver J (2018). The biology of regeneration failure and success after spinal cord injury. Physiol Rev.

[47] Chen Z, Guo H, Lu Z, Sun K, Jin Q (2019). Hyperglycemia aggravates spinal cord injury through endoplasmic reticulum stress mediated neuronal apoptosis, gliosis and activation. Biomed Pharmacother.

[48] Li X, Yu Z, Zong W, Chen P, Li J, Wang M, Ding F, Xie M, Wang W, Luo X (2020). Deficiency of the microglial Hv1 proton channel attenuates neuronal pyroptosis and inhibits inflammatory reaction after spinal cord injury. J Neuroinflammation.

[49] Zhang X, Dong H, Li N, Zhang S, Sun J, Zhang S, Qian Y (2016). Activated brain mast cells contribute to postoperative cognitive dysfunction by evoking microglia activation and neuronal apoptosis. J Neuroinflammation.

[50] Block ML, Hong J (2005). Microglia and inflammation-mediated neurodegeneration: multiple triggers with a common mechanism. Prog Neurobiol.

[51] Orr MB, Gensel JC (2018). Spinal cord injury scarring and inflammation: therapies targeting glial and inflammatory responses. Neurotherapeutics.

[52] Bradbury EJ, Burnside ER (2019). Moving beyond the glial scar for spinal cord repair. Nat Commun.

[53] Gaudet AD, Fonken LK (2018). Glial cells shape pathology and repair after spinal cord injury. Neurotherapeutics.

[54] Leibinger M, Zeitler C, Gobrecht P, Andreadaki A, Gisselmann G, Fischer D (2021). Transneuronal delivery of hyper-interleukin-6 enables functional recovery after severe spinal cord injury in mice. Nat Commun.

