# Supplementary figures and images for: FANCC deficiency mediates microglial pyroptosis and secondary neuronal apoptosis in spinal cord contusion
Source: Cell Biosci. 2022 Jun 3;12:82. doi: 10.1186/s13578-022-00816-4 (PMC9164466; doi:10.1186/s13578-022-00816-4)

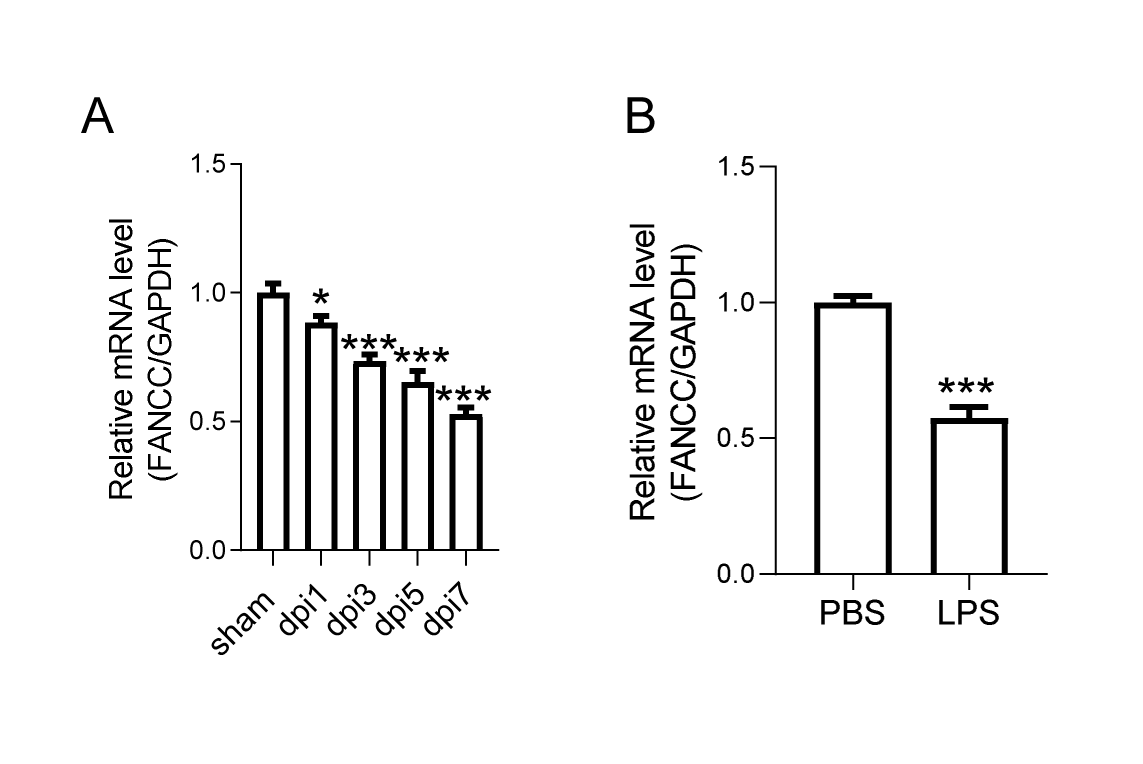

Supplement: Supplementary file 1 — Additional file 1: Figure S1. A Relative mRNA level of FANCC in the spinal cord within a week post-injury; n = 5. The error bars represent the SD. *p < 0.05 vs. Sham group by one-way ANOVA followed by Tukey’s post hoc analysis (*p < 0.05, **p < 0.01, and ***p < 0.001). B Relative mRNA level of FANCC in LPS-stimulated primary microglia for 12h; n = 5. The error bars represent the SD. *p < 0.05 vs. PBS group by t-test (*p < 0.05, **p < 0.01, and ***p < 0.001). [file 13578_2022_816_MOESM1_ESM.tif]

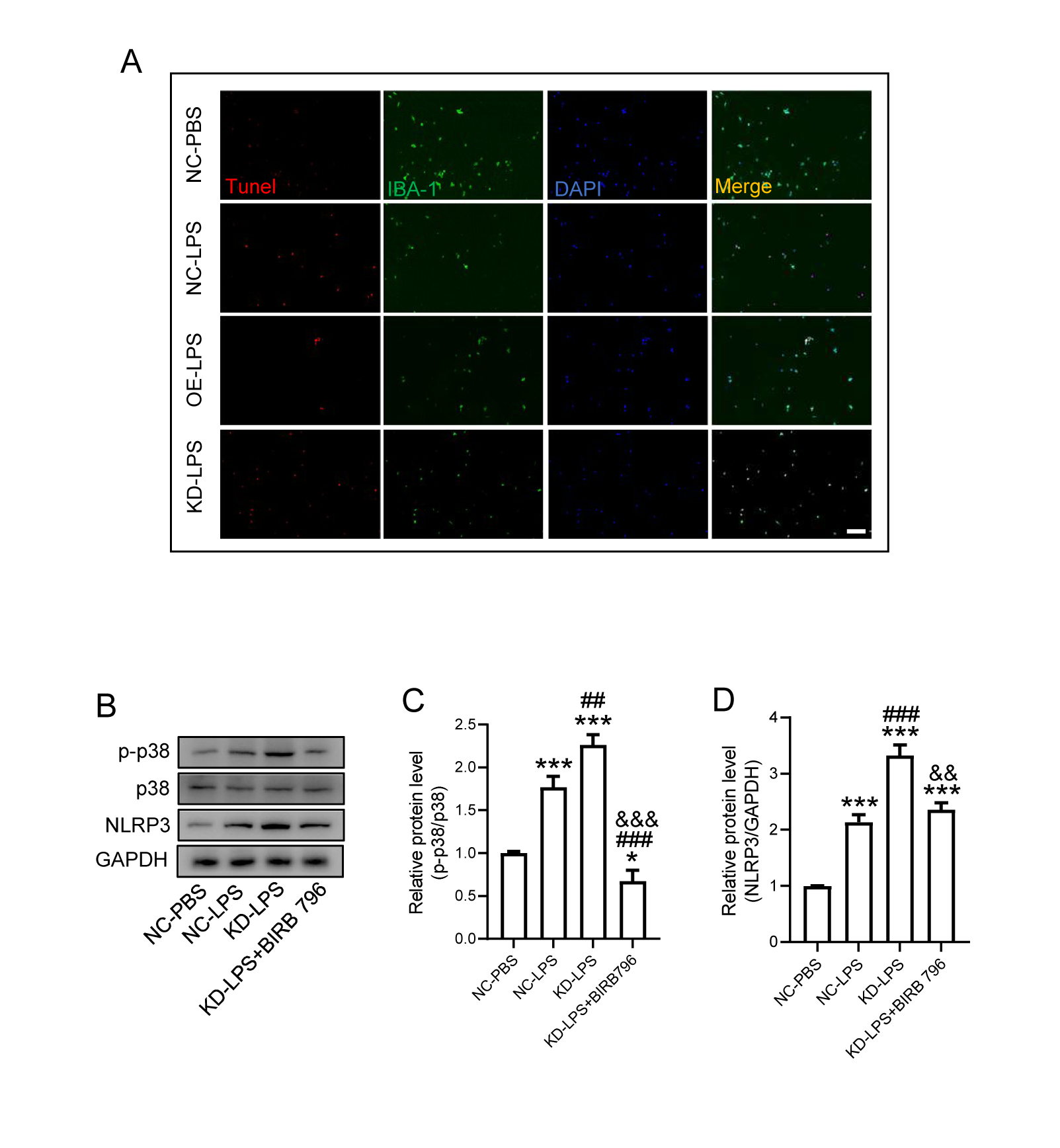

Supplement: Supplementary file 2 — Additional file 2: Figure S2. A Microglial death determined by TUNEL assay in LPS-activated microglia after transfection with OE-FANCC and KD-FANCC; Scale bar = 200 μm. B Western blotting performed for p-p38, p38 and NLRP3 in LPS-activated microglia pretreated with BIRB 796 after transfection with KD-FANCC; n = 3. GAPDH was used as the control. C Bar graph showing the ratio analysis of p-p38/p38. D Densitometric analysis of NLRP3 expression. The error bars represent the SD. *p < 0.05 vs. NC-PBS group, #p < 0.05 vs. NC-LPS group, &p vs. KD-LPS group by one-way ANOVA followed by Tukey's post hoc analysis (*p < 0.05, **p < 0.01, and ***p < 0.001). [file 13578_2022_816_MOESM2_ESM.tif]
